# Investigations on the Viscoelastic Performance of Pressure Sensitive Adhesives in Drug-in-Adhesive Type Transdermal Films

**DOI:** 10.1007/s11095-014-1318-2

**Published:** 2014-03-06

**Authors:** Hans-Michael Wolff, Irsan, Kalliopi Dodou

**Affiliations:** 1 UCB Biosciences GmbH, Alfred-Nobel-Str. 10, 40789 Monheim, Germany; 2 Sunderland Pharmacy School, Faculty of Applied Sciences, University of Sunderland, Science Complex, Wharncliffe Street, SR1 3D Sunderland, UK

**Keywords:** acrylic adhesive, rheology, silicone adhesive, solubility parameter, transdermal patch

## Abstract

**Purpose:**

We aimed to investigate the effect of solubility parameter and drug concentration on the rheological behaviour of drug-in-adhesive films intended for transdermal application.

**Methods:**

Films were prepared over a range of drug concentrations (5%, 10% and 20% w/w) using ibuprofen, benzoic acid, nicotinic acid and lidocaine as model drugs in acrylic (Duro-Tak 87-4287 and Duro-Tak 87900A) or silicone (Bio-PSA 7-4301 and Bio-PSA 7-4302) pressure sensitive adhesives (PSAs). Saturation status of films was determined using light microscopy. Viscoelastic parameters were measured in rheology tests at 32°C.

**Results:**

Subsaturated films had lower viscoelastic moduli whereas saturated films had higher moduli than the placebo films and/or a concentration-dependent increase in their modulus. Saturation concentration of each drug in the films was reflected by decreasing/increasing viscoelastic patterns. The viscoelastic windows (VWs) of the adhesive and drug-in-adhesive films clearly depicted the effect of solubility parameter differences, molar concentration of drug in the adhesive film and differences in PSA chemistry.

**Conclusions:**

Drug solubility parameters and molar drug concentrations have an impact on rheological patterns and thus on the adhesive performance of tested pressure sensitive adhesives intended for use in transdermal drug delivery systems. Use of the Flory equation in its limiting form was appropriate to predict drug solubility in the tested formulations.

## INTRODUCTION

Transdermal Drug Delivery (TDD) systems, commonly also known as transdermal patches, are pharmaceutical dosage forms intended to deliver drug across a patient’s skin into the systemic circulation at a therapeutically effective rate. Since the market introduction of transdermal patches, it has become evident that optimisation of their adhesive properties represents an important challenge during the pharmaceutical development in order to minimise the risk of adhesion failure in practice (1).

Adhesion is critical to the safety, efficacy and quality of TDD products. There have been numerous reports to the Food and Drug Administration (FDA) on problems caused by the lack of adhesion of patches to the skin. Therapeutic effect is correlated to adhesive performance therefore drug delivery from TDD systems will be reduced if the surface area of contact decreases (e.g. patch lift), leading to incorrect dosing. In addition, the cost of treatment increases if adhesive failure occurs during the prescribed application time because the patient will have to apply a new patch. Safety issues can also arise e.g. children accidentally dosed on picking up fallen patches, or by sitting or lying on a fallen patch (2).

The pressure sensitive adhesive (PSA) is the material that confers to the transdermal patch its adhesive ability. PSAs are permanently tacky adhesive polymers that can stick to a substrate by application of light pressure (stress) without requiring solvent or heat. There are several possible patch designs. With respect to main structural elements and intrinsic release kinetics one may distinguish between reservoir- and matrix-type systems. In our study we examined the properties of drug-in-adhesive patches, i.e. matrix systems, in which the drug is mixed directly with the adhesive polymer.

The European Medicines Agency has recently drafted a guidance document on the quality of transdermal patches (3). In this document the importance of *in vitro* adhesion tests is highlighted and defined as the characterisation of the adhesive/cohesive and viscoelastic properties of the patch. In routine standard tests, the adhesive performance of transdermal patches and their batch-to-batch uniformity is evaluated using non physiological substrates, e.g. the patch is placed on a metal plate to determine its peel adhesion properties. In this work, the adhesive performance is evaluated by mainly focussing on the material properties manifested by the rheological properties of the PSA. Advantageously, such measurements do not depend on surface properties of non-physiological substrates used in routine standard tests but purely on the material properties; hence the variation from metal surface is eliminated.

PSAs are viscoelastic in nature i.e. they can behave both as viscous liquids and elastic solids; during application to a substrate, PSAs behave as a viscous liquid layer that spreads onto the substrate. The spreading is achieved by light pressure when applying the PSA hence the name ‘pressure sensitive adhesive’. During removal from the substrate, the PSA behaves as a cohesive solid to ensure complete removal without leaving any residues. The first PSAs were made from the addition of resin to natural rubber at appropriate ratios that would render pressure sensitive adhesive performance to the system (4). The tackifier resin provides the liquid behaviour by decreasing the shear modulus at slow deformation rate when wetting happens. The solid behaviour leads to an increase in the modulus at higher rates when the system is removed from the substrate. By having dual properties, PSAs require good compromise between adhesion (surface wetting) and cohesion (bearing load) balance.

The first approach to relate the adhesive performance of pressure sensitive adhesives to their rheological properties can be attributed to the work of Dahlquist (5) who suggested that the creep compliance J of the adhesive has to be greater than 10^−5^ Pa^−1^ at low frequencies that correspond to the bonding process. Subsequently, Chu expanded the work of Dahlquist by a detailed and thorough analysis using dynamic rheology testing instead of Dahlquist’s static test: Chu found that rubber PSAs tested by him revealed an optimum combination of tack, shear and peel properties when their elastic modulus G′ measured at different frequencies met the following criteria:

1. G′ at ω = 0.1 rad/sec = 2 to 4 × 10^4^ Pa = 2 to 4 N/cm^2^
2. Slope G′(at ω = 100 rad/sec)/G′(at ω = 0.1 rad/sec) = 5 to 300 (6).

Chang further expanded the work of Dahlquist and Chu by introducing a two dimensional box, forming a viscoelastic window (VW). The VW is constructed via four coordinates that correspond to the measured elastic (G′) and viscous (G″) moduli at two frequencies; at 0.01 rad/sec (low frequency) corresponding to bonding and at 100 rad/sec (high frequency) corresponding to debonding. Chang subsequently constructed the VWs of several PSAs and developed VW areas that correlate to the adhesion performances of different types of PSAs (7,8). A correlation between traditional tape properties and rheological data and consistency with Chu’s criteria has already been reported for silicone-type PSAs (9).

In this study we selected a number of low molecular weight model drugs to investigate the impact of drug load on rheological properties of two classes of pressure sensitive adhesive (PSA) polymers (acrylic- and silicone-type PSAs) suitable for transdermal systems. Previous studies on saturated drug-in-adhesive silicone films had shown a drug concentration-dependent increase on the moduli of the films (10). In this current work we examine both subsaturated and saturated films. The selected silicone-type PSAs have the same chemical composition but different solvents (ethyl acetate or n-heptane), whereas the tested non cured acrylic-type PSAs represent chemically different copolymers in the same solvent (ethyl acetate).

The aims of our study were:

- To study how drug load and drug solubility parameter affect the rheological properties of pressure sensitive adhesives.
- To correlate the rheological properties of pressure sensitive adhesives and drug-in-adhesive films with existing criteria that describe adhesive performance.
- To examine whether pre-formulation information on drug-in-adhesive films can be utilised to predict their adhesive performance.

## MATERIALS AND METHODS

### Materials

Two acrylic and two silicone PSAs were used in our study. Duro-Tak 87-900A and Duro-Tak 87-4287, both acrylic non cured PSAs in ethyl acetate, were supplied by National Adhesives-Henkel (Slough, UK). Bio-PSA 7-4301, an amine compatible silicone PSA in n-heptane and Bio-PSA 7-4302, an amine compatible silicone PSA in ethyl acetate were supplied by Dow Corning Corporation (Midland, Michigan, USA). Scotchpak 1020, a low adhesion release liner, was supplied by 3 M Corporation (St Paul, USA). Four model drugs were selected; nicotinic acid (Fluka, Belgium), ibuprofen (Knoll Pharmaceuticals, BASF, UK), lidocaine (Sigma, Taiwan) and benzoic acid (Riedel-de Haen, Sigma-Aldrich, Germany). Ethyl acetate (purity of 99.7%) was supplied by Sigma-Aldrich (Steinheim, Germany) and toluene (purity of 99.7%) was supplied by Riedel-de Haen (Seelze, Germany).

### Methods

#### Calculation of Solubility Parameters of Model Drugs, Solvents and Monomer Units

Solubility parameter components of solvents, acrylic and silicone monomer structures and model drugs were calculated according to the methods of Hoftyzer and Van Krevelen (11) and Fedors (12). The molar volume data was referred from Fedors (12). The acrylic monomer structures were drawn as if they were in the respective polymer, by changing their double bond (=CH_2_) to a single bond (-CH_2_-) prior to the calculation of solubility parameter, to reflect the real structures of the repeating monomer units as they are arranged by the polymerisation process. Similarly, the silanol groups (Si-OH) of the silicone monomers were drawn in their siloxane form (Si-O). The compositions of Duro-Tak 87-900A and Duro-Tak 87-4287 were taken from patent publications (13–16). Both Duro-Tak 87-900A and Duro-Tak 87-4287 have 2-ethylhexyl acrylate as the main repeating monomer unit. Duro-Tak 87-4287 is a copolymer with vinyl acetate and contains OH– functional groups as 2-hydroxyethyl acrylate is also part of the polymer composition. Duro-Tak 87-900A contains besides 2-ethylhexyl acrylate, butylacrylate, methyl methacrylate and tertiary-octyl acrylamide units. Bio-PSA 7-4301 and Bio-PSA 7-4302 contain MQ silicate resin (Q = SiO_4/2_; M = R^1^R^2^R^3^SiO_1/2_; R = CH_3_ or OH) and PDMS poly (dimethyl siloxane) silicone polymer at a ratio of 55:45 (17).

**Fig. 1:**
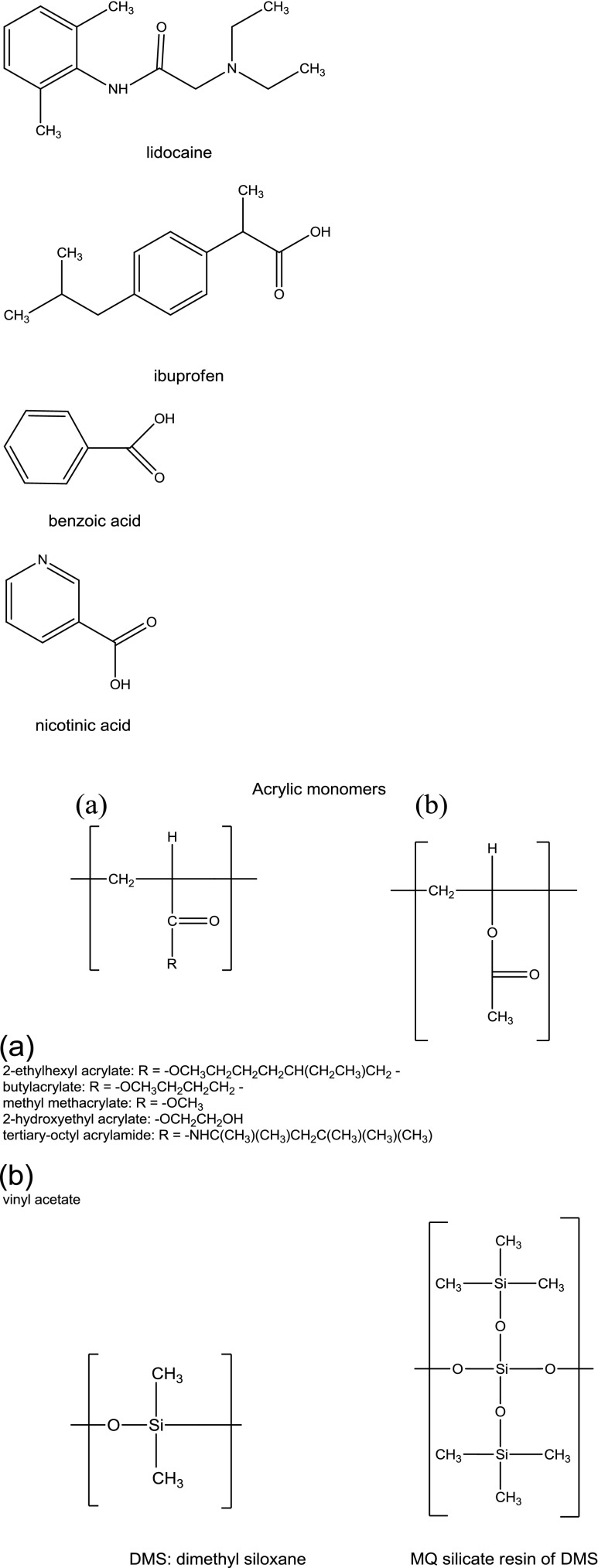
Structures of model drugs and PSA monomers.

The chemical structures of the model drugs and monomer units are shown on Fig. 1.

The following equations were used for the calculation of solubility parameter δ according to Hoftyzer and Van Krevelen’s method:

1 $$ {\delta}_d=\frac{{\displaystyle \sum {F}_{di}}}{v} $$

where *δ*_*d*_ is the solubility parameter contribution of dispersion components, F_di_ is the  group contribution to the dispersion components, and V is the  molar volume.

2 $$ {\delta}_p=\frac{\sqrt{{\displaystyle \sum {F}_{pi}^2}}}{v} $$

where δ_p_ is the solubility parameter contribution of polar components, F_pi_ is the group contribution to the polar components, and V is the molar volume.

3 $$ {\delta}_h=\sqrt{\frac{{\displaystyle \sum {E}_{hi}}}{v}} $$

where δ_h_ is the solubility parameter contribution of hydrogen bonding components, E_hi_ is the hydrogen bonding energy, and V is the molar volume.

4 $$ {\delta}_t=\sqrt{\delta_d^2+{\delta}_p^2+{\delta}_h^2} $$

where δt is the total solubility parameter.

5 $$ {\delta}_A=\sqrt{\delta_p^2+{\delta}_h^2} $$

where δ_A_ is the overall polarity.

The following equation was used for the calculation of solubility parameters using Fedors’ method:

6 $$ \delta =\sqrt{\frac{{\displaystyle \sum energy\kern0.5em  of\kern0.5em  vaporisation}}{{\displaystyle \sum molar\kern0.5em  volume}}} $$

where δ is the total solubility parameter.

#### Determination of Solid Content of Liquid Adhesives

Approximately 3 ml of liquid adhesive (Duro-Tak 87-900A, Duro-Tak 87-4287, Bio-PSA 7-4301 or Bio-PSA 7-4302) was pipetted into a previously tarred beaker and weighed accurately using an analytical balance. Drying of the solvent took place inside an oven (Memmert Model UNE 400, Schwabach, Germany) at 40 ± 1°C for 2 h. The beaker was then left at ambient room temperature for an hour before being weighed again. The process was conducted in duplicate (*n* = 2) for each pressure sensitive adhesive. The solid content of each liquid adhesive was then determined as a percentage. Results were verified by examination of residual solvent using Headspace Gas Chromatography as described in the following section. The mean value of solid content was subsequently used for the calculation of the required amount of liquid adhesive for each target% w/w drug load in the dry drug-in-adhesive films.

#### Residual Solvent Analysis Using Headspace Gas Chromatography

Residual solvent in the dry adhesive was determined by Headspace Gas Chromatography (GC) (Agilent Technologies 7890A, Santa Clara, CA, USA) using an internal standard of toluene/N,N-dimethyl acetamide. A calibration curve was prepared, using five solutions of n-heptane/ethyl acetate in internal standard, for the quantification of n-heptane and ethyl acetate in the dry adhesive samples. An accurately weighed adhesive sample was placed into a 20 ml headspace vial containing 5 ml of internal standard. The vial was then capped immediately. Prior to calibration, a blank of internal standard was injected. All adhesive samples were examined in triplicate (*n* = 3).

A DB-624, 30 m × 0.53 mm × 3.0 μm GC column was used with Helium gas as the carrier (constant flow of 35 cm/min) with 3.5 psi gas pressure. The United States Pharmacopoeial method, USP 467, for the Headspace analysis of organic volatile impurities was applied. (18)

#### Preparation of Drug Loaded Adhesive Films

Drug-in-adhesive films with drug loading of 5%, 10% and 20% w/w were prepared in triplicate (*n* = 3). About 15 ml of liquid adhesive was pipetted into a previously tared glass container. The container was quickly closed with its cap to minimise evaporation of the solvent. The amount of model drug needed was then calculated and added into the glass container. Another cap which had been drilled to accommodate the shaft of an anchor stirrer (IKA, Staufen, Germany) was quickly secured to the glass container and mixing was then carried out at 65 ± 3 rpm for 10 min using an overhead stirrer (IKA, Staufen, Germany).

A siliconized polyester foil was cut to the required length to fit the Erichsen Model 509/1 film coater (Hemer-Sundwig, Germany) with the release coating side of the polyester-liner facing up. The solvent-containing drug loaded adhesive mass preparations were coated onto the polyester liner sheets. The film coater was previously set to be horizontal by adjusting the base of the coater against a spirit level. The knife blade was set to the height of 0.60 mm by adjusting the two height adjusters on the blade against a 0.60 mm feeler gauge blade (DIN 2275) inserted between the film coater base and the knife blade. An oil-less vacuum pump (FB 70155, Fisherbrand) was connected to the base of the coater to give a vacuum that ensured the release liner was flat. The speed of the blade was set to 6 mm/sec. The film was then dried at 40°C for 120 min using a universal oven (Memmert Model UNE 400, Schwabach, Germany) controlled via a computer running Celsius 2005 software (version 6.1). The oven was previously set to be horizontal by adjusting the base of the oven against a spirit level. The film was laminated with another layer of release liner as soon as it came out of the oven. It was kept at room temperature and used for rheological analysis within 3 weeks.

Unmedicated (placebo) adhesive preparations were also coated in triplicate for each PSA using the above method.

#### Light Microscopy

Polarised light microscopic examination of all drug-in-adhesive films was conducted using an Olympus, BH-2 microscope (Optivision Ltd, Japan) fitted with a 10× lens, a camera (AxioCam, MRc, Carl Zeiss) and image acquisition software (AxioVision, vs4.4., Carl Zeiss). The drug loaded adhesive films, coated with release liners on both sides, were observed under the microscope with the polariser set at 45°.

#### Estimation of Drug Solubility in the Films

An estimate of equilibrium solute solubility was calculated for the model drugs, using the limiting form of the Flory equation expressed as shown in Eq. 7 (19):

7 $$ {\phi}_1= \exp -\left[1+{\chi}_1\right]= \exp -\left[1.34+\frac{\nu_1}{ RT}\times {\left({\delta}_1-{\delta}_2\right)}^2\right] $$

where Φ_1_ is the volume fraction of the solute (=drug) ≈ drug mass fraction, Χ_1_ is the interaction parameter =

8 $$ \left[0.34+\frac{\nu_1}{ RT}\times {\left({\delta}_1-{\delta}_2\right)}^2\right] $$

ν_1_ is the molar volume of the drug, δ_1_ is the solubility parameter of the drug, δ_2_ is the solubility parameter of the polymer, R is the gas constant (8.314 J × K^−1^ mol^−1^), and T is the selected temperature (K) = 298 K.

It has to be noted that this equation is valid only if any specific molecular interactions between drug and polymer segments like e.g. acid base reactions or formation of defined drug-polymer complexes can be neglected.

#### Rheological Tests

The rheological measurements of placebo and drug loaded adhesive films were performed on a Bohlin Gemini 200 Advanced Rheometer (Malvern Instruments, Malvern, UK) attached to a water bath (Julabo F12-MC, Seelbach, Germany) and an oil-less compressor (Jun-Air 2000, Norresundby, Denmark). Film samples were placed between an 8 mm diameter stainless steel serrated upper parallel plate and serrated lower plate. The gap size was set to 1,000 μm. All tests were performed in triplicate at 32 ± 0.1°C, i.e. in the temperature range of human skin *in vivo*.

##### Amplitude Sweep

Prior to running frequency sweep and time temperature superposition tests, an amplitude sweep within a stress range from 2 to 20,000 Pa was carried out in triplicate at a fixed frequency of 1 Hz to define the linear viscoelastic region (LVR). Frequency sweep test was subsequently carried after setting appropriate stress and strain values which were within the LVR.

##### Frequency Sweep

Frequency sweeps were conducted for each sample at decreasing frequencies from 100 rad/sec down to 0.1 rad/sec. The mean elastic modulus (G′) and viscous modulus (G″) at 0.1 rad/and 100 rad/sec were recorded for each sample.

##### Creep and Recovery

Creep tests were performed with 1800 s of creep time and 1800 s recovery time. The constant shear stress was set to 1,000 Pa. The mean creep compliance (J) at 1800 s was noted for analysis.

#### Plotting of Viscoelastic Data in Comparison to Known Assessment Criteria

Elastic moduli were plotted against viscous moduli at 0.1 and 100 rad/sec in log scale and compared with known acceptance criteria for PSAs. A slight modification was made to the central viscoelastic window of Chang (7) by measuring *G*′ & *G*″ at 0.1 rad/sec instead of 0.01 rad/sec, to reduce the time requirements for the measurements, also considering that test temperature (32°C) was also slightly increased, and that a temperature increase has qualitatively the same impact on the shear modulus as a decrease of the deformation frequency.

#### Statistical Analysis

All statistical calculations were done using SPSS 17.0 (SPSS UK Ltd., Woking, UK). One way analysis of variance (ANOVA) was used to analyse the frequency sweep and creep data. One-sample *t*-test was used to determine the significance of deviation from Chu’s criteria. A probability of *p* <0.05 was taken as significant difference. The Scheffe method was used as ANOVA post hoc analysis.

## RESULTS

### Solubility Parameters of Model Drugs and Monomer PSA Units

The calculated solubility parameter values for the model drugs are shown in Table I alongside their physicochemical properties. Both methods (Fedors and Van Krevelen) gave very similar results in their total solubility parameter. Besides, the model drugs covered a wide range of solubility parameter values.

**Table I:** Physicochemical Properties and Calculated Solubility Parameter Values for the Model Drugs

Model compound	Molecular weight (Dalton)	Density g/cm^3^	Molar volume cm^3^/g	Total δ (MPa^1/2^) Fedors method	Total δ (MPa^1/2^) Hoftyzer &Van Krevelen method	δ_A_	% δ_A_/δ
Lidocaine	234.3	1.03	228.4	19.90	19.29	8.01	41.5
Ibuprofen	206.3	1.18	175.6	20.91	19.36	7.49	19.36
Benzoic acid	122.1	1.27	96.5	24.41	22.45	10.91	22.45
Nicotinic acid	123.1	1.47	83.7	28.01	28.87	19.93	28.87

The solubility parameter values of the adhesive monomer units and the solvents are presented in Tables II. Table III shows the difference in solubility parameter between each drug and the main adhesive monomer unit of each PSA and the predicted drug solubility according to the Flory equation (see section below).

**Table II:** Total Solubility Parameters of Adhesive Monomer Units and Solvents Calculated Using Hoftyzer & Van Krevelen and Fedors Methods

Monomer unit	Adhesive	Total δ (MPa^1/2^) (Fedor’s method)	Total δ (MPa^1/2^) (Hoftyzer & Van Krevelen method)
2-ethylhexyl acrylate	*Duro-Tak 87-900A;* *Duro-Tak 87-4287*	18.86	18.09
Butyl acrylate	*Duro-Tak 87-900A*	19.98	19.32
Methyl methacrylate	*Duro-Tak 87-900A*	20.32	20.64
Tertiary-octyl acrylamide	*Duro-Tak 87-900A*	19.31	18.64
2-hydroxyethyl acrylate	*Duro-Tak 87-4287*	27.23	28.05
Vinyl acetate	*Duro-Tak 87-4287*	21.60	21.51
DMS (dimethyl siloxane)	*Bio-PSA 7-4301* *Bio-PSA 7-4302*	15.10	–
Silicate Resin for DMS	*Bio-PSA 7-4301* *Bio-PSA 7-4302*	15.48	–
Solvent	–		
Ethyl acetate	–	17.89	17.69
n-heptane	–	15.20	14.85

**Table III:** Total Solubility Parameter Difference Between Model Drugs and Main Monomers of Duro-Tak and Bio-PSA and Predicted Drug Solubility at Equilibrium Estimated from the Volume Fraction of the Limiting Form of the Flory Eq. 7 (18)

Model drug	Total δ (MPa^½^) (Fedors method)	Total δ difference with 2-ethylhexyl acrylate	Total δ difference with DMS	Predicted drug solubility (%) Acrylic PSA Silicone PSA	
Lidocaine	19.90	1.04	4.80	23.7	3.1
Ibuprofen	20.91	2.05	5.80	19.4	2.4
Benzoic acid	24.41	5.55	9.31	7.9	0.9
Nicotinic acid	28.01	9.15	12.91	1.5	0.1

### Estimation of Drug Solubility in Films Using Modified Form of Flory’s Equation

Using the limiting form of Flory’s equation (Eq. 7), the solubility of all model drugs was estimated to be higher in acrylic PSAs than in silicone PSAs (Table II). Lidocaine had the highest estimated solubility whereas nicotinic acid had the lowest estimated solubility in both acrylic and silicone PSAs.

### Residual Solvent Analysis

Retention times of ethyl acetate and heptane were 2.3 min and 3.1 min respectively. Residual content analysis in the dry adhesive samples during the solid content determination showed: 0.51 ± 0.19% residual ethyl acetate in BIO-PSA 4302; 0.62 ± 0.13 residual n-heptane in BIO-PSA 4301; 3.73 ± 0.73% residual ethyl acetate in Duro-Tak 87-4287; 6.52 ± 1.06% residual ethyl acetate in Duro-Tak 87-900A. Residual solvent in all manufactured placebo and drug loaded films was negligible, less than 0.1%. For example, residual ethyl acetate was 0.029 ± 0.003% in Duro-Tak 87-4287 placebo film and 0.015 ± 0.007 in Duro-Tak 87-4287 film containing 20% w/w benzoic acid.

### Polarised Microscopy

Over the test time period of at least 3 weeks at uncontrolled room temperature benzoic acid remained crystal-free at 5% w/w load in both Duro-Tak 87-900A and Duro-Tak 87-4287; whereas at 10% w/w load, it was soluble only in Duro-Tak 87-900A and at 20% w/w it was suspended in both Duro-Taks. Lidocaine and ibuprofen were soluble in both acrylic polymers, particularly in Duro-Tak 87-900A, whereas they were suspended in the silicone adhesives. Nicotinic acid was suspended at all tested concentrations in both the acrylic-type (Duro-Tak) and silicone-type (Bio-PSA) adhesives.

### Rheological Measurements

#### Frequency Sweep

**Fig. 2:**
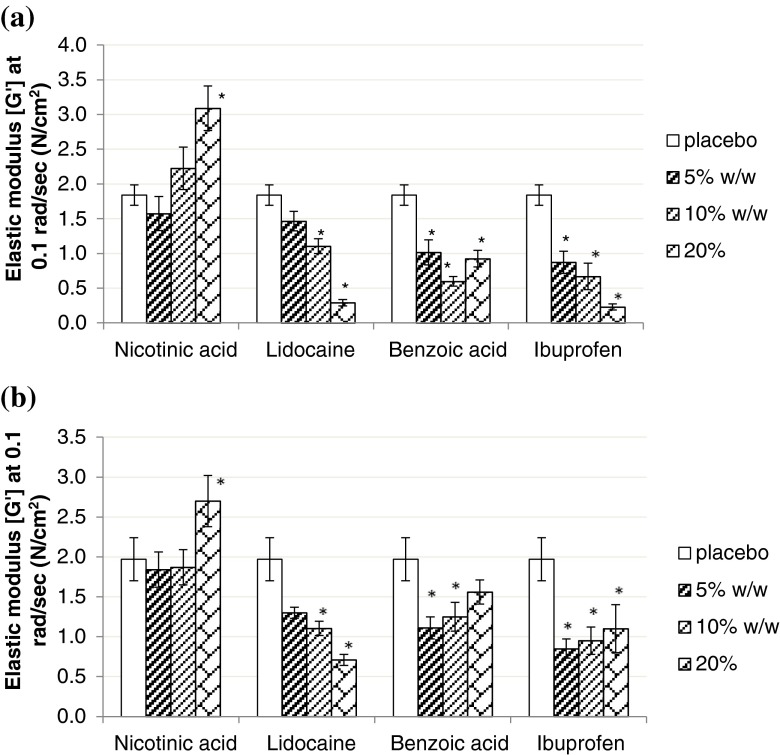
Elastic modulus (G′) at 0.1 rad/sec of the model drugs at nominal 5%, 10% and 20% w/w drug load ** in (**a**) Duro-Tak 87-900A and (**b**) Duro-Tak 4287, compared to their respective placebo films (*n* = 3). *Statistically significant difference (*p* < 0.05) from the neat adhesive (placebo). **Related to the nominal concentrations drug load could be approximately 5% higher for Duro-Tak 4287 and 12% higher for Duro-Tak 87-900A considering solvent residues measured on adhesive solids.

Films loaded with lidocaine, ibuprofen and benzoic acid, had significantly lower elastic moduli compared to their respective placebo films. This observation was more pronounced in the acrylic-type PSAs (Duro-Tak) than in silicone-type PSAs (Bio-PSAs). It was especially pronounced in Duro-Tak 87-900A (Fig. 2a).

##### Duro-Tak 87-900A and Duro-Tak 87-4287

**Fig. 3:**
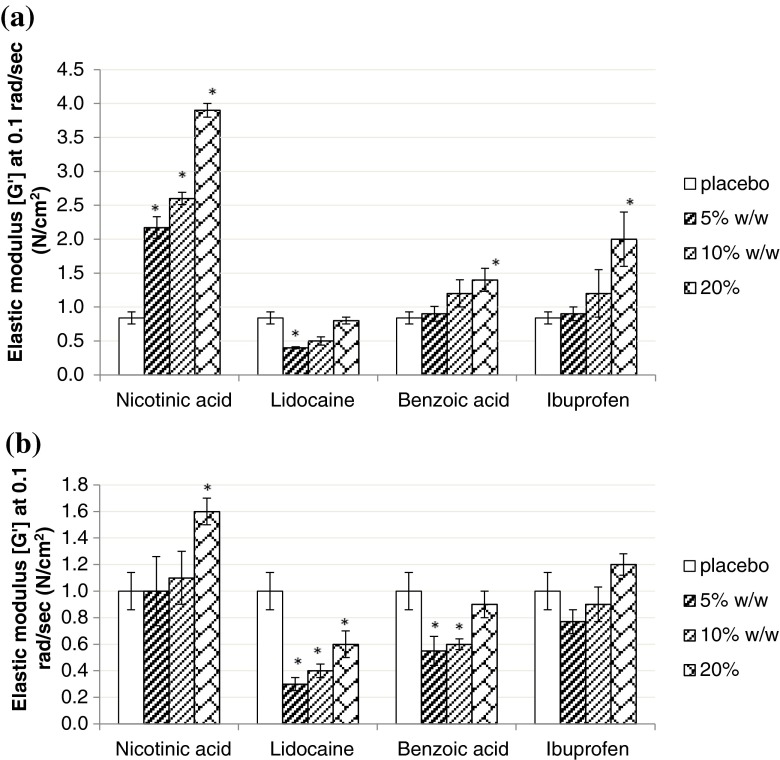
Elastic modulus (G′) at 0.1 rad/sec of the model drugs at nominal 5%, 10% and 20% w/w drug load ** in (**a**) Bio-PSA 7-4302 and (**b**) Bio-PSA 7-4301 compared to their respective placebo films (*n* = 3). *Statistically significant difference (*p* < 0.05) from the neat adhesive (placebo). **Related to the nominal concentrations drug load could be approximately 0.5% higher for Bio-PSA 7-4302 and 1% higher for Bio-PSA 7-4301 considering solvent residues measured on adhesive solids.

The elastic modulus (G′) at 0.1 rad/sec of all model drugs in Duro-Tak 87-900A is shown in Fig. 3a. Lidocaine and ibuprofen loaded films showed a decrease in the elastic modulus (G′) with increasing drug load whereas nicotine loaded films showed an increase in the elastic modulus (G′) with increasing drug load. For benzoic acid loaded films, the elastic modulus (G′) decreased from 5 to 10% w/w whereas it increased from 10 to 20% w/w drug load. The elastic modulus (G′) at 0.1 rad/sec of all model drugs in Duro-Tak 87-4287 is shown in Fig. 2b. With increasing drug load, only lidocaine showed a decrease in the elastic modulus (G′) while the rest of the model drugs showed an increase in the elastic modulus (G′).

##### Bio-PSA 7-4302 and Bio-PSA 7-4301

The elastic modulus (G′) at 0.1 rad/sec in Bio-PSA 7-4302 and Bio-PSA 7-4301 is shown for all model compounds in Fig. 3a and b respectively. In both silicone-type adhesives, G′ values consistently increased with increasing drug concentrations from 5 to 20% w/w.

In Bio-PSA 7-4302 only the G′ values of lidocaine loaded films remained below the G′ of the placebo preparation indicating a higher solubility of this compound in the silicone adhesive matrix. In Bio-PSA 7-4301, the moduli of all drug loaded films were lower than in Bio-PSA 7-4302. Also, the moduli of all drug loaded Bio-PSA 7-4301 films, except with nicotinic acid, were higher than the placebo’s.

#### Creep Test

**Fig. 4:**
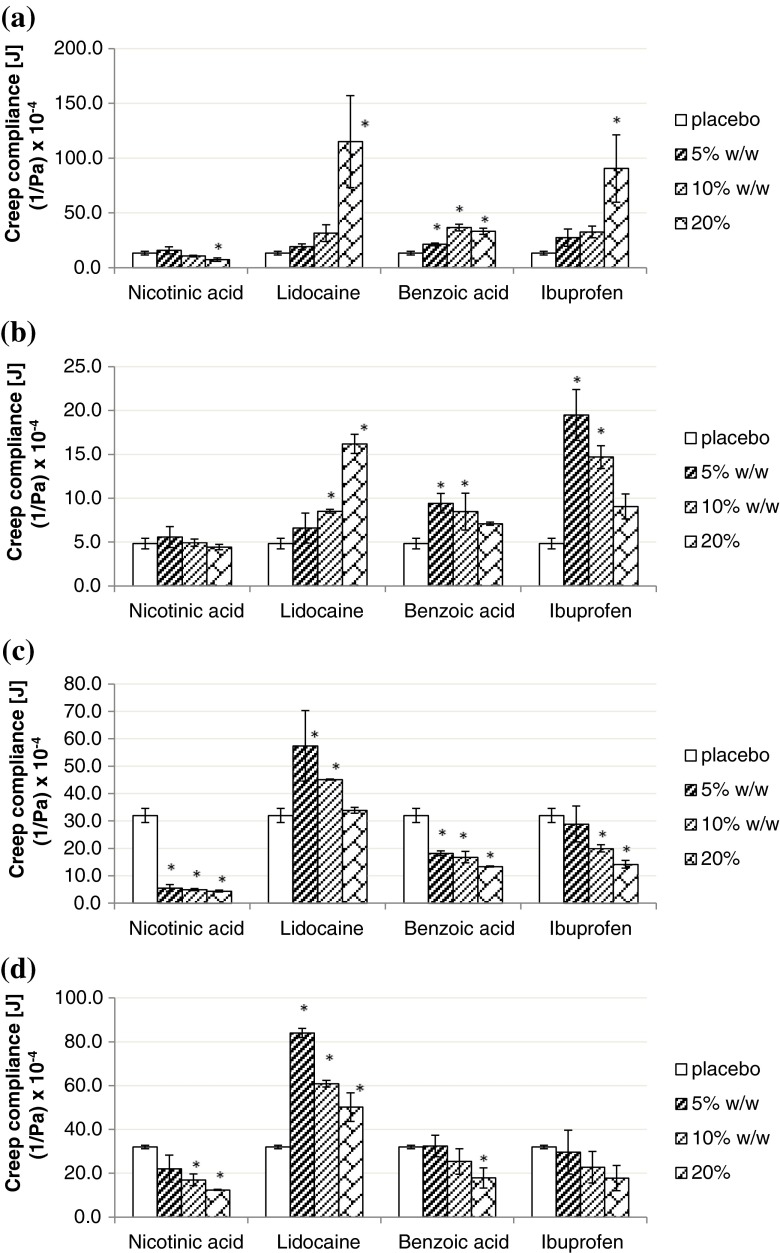
Creep compliance of placebo and model drugs at nominal 5%, 10% and 20% w/w in (**a**) Duro-Tak 87-900A; (**b**) Duro-Tak 87-4287; (**c**) Bio-PSA 4302 and (**d**) Bio-PSA 4301 (*n* = 3). *Statistically significant difference (*p* < 0.05) from the neat adhesive (placebo).

Creep compliance of Duro-Tak 87-900A loaded with lidocaine and ibuprofen was increasing proportionally with drug concentration from 5% w/w to 20% w/w (Fig. 4a). For benzoic acid the creep compliance showed an increasing trend up to 10% w/w and then a decreasing trend when the concentration was further increased to 20% w/w. For nicotinic acid, creep compliance values were decreasing as the drug concentration was increased. Creep compliance of lidocaine in Duro-Tak 87-4287 films (Fig. 4b) was increasing proportionally with drug concentration from 5% w/w to 20% w/w. An opposite trend was observed on ibuprofen, benzoic acid and nicotinic acid. Similar to Duro-Tak 87-900A, films of Duro-Tak 87-4287 loaded with lidocaine, ibuprofen and benzoic acid had a statistically higher creep compliance compared to placebo films. A decreasing trend in the creep compliance of all model drugs as the drug concentration was increased from 5% w/w to 20% w/w was observed in all Bio-PSA 4301 and Bio-PSA 4302 films (Fig. 4c and d). Films loaded with lidocaine, ibuprofen and benzoic acid (with solubility parameters similar to the adhesive monomer units) had a relatively higher creep compliance compared to placebo films.

The creep compliance of Duro-Tak 87-900A (13.2 × 10^−4^ Pa^−1^) was approximately three times that of Duro-Tak 87-4287 placebo (4.8 × 10^−4^ Pa^−1^). Both silicone-type PSAs showed greater creep compliance (32 × 10^−4^ Pa^−1^) than the acrylic-type PSAs.

#### Chu’s Criteria and Viscoelastic Windows (VWs)

**Fig. 5:**
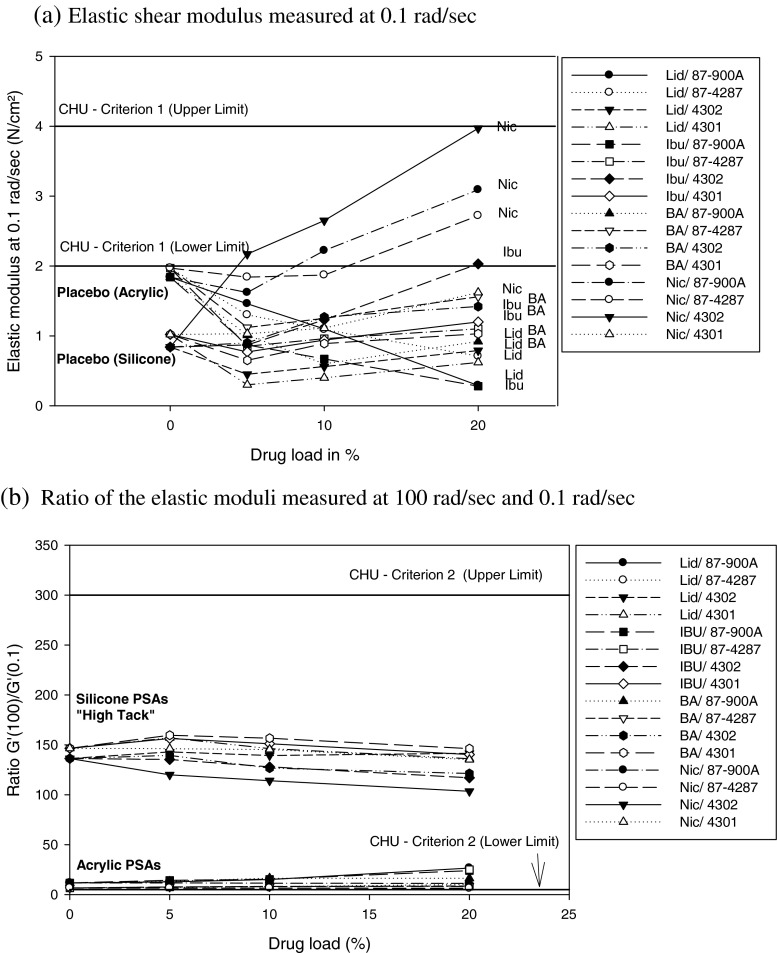
Viscoelastic properties of tested samples relating to CHU’s criteria.

Figures 5a and b illustrate the rheological results with respect to Chu’s criteria. All films passed Chu’s 2nd criterion, ratio [G′ (at ω = 100 rad/sec)/G′ (at ω = 0.1 rad/sec)]. All silicone PSAs with and without drug were located in the mid of the target region in contrast to acrylic-type films which were found to be near the lower limit of the 2nd acceptance criterion. For the majority of test samples there was discordance with Chu’s 1st criterion; this finding is consistent with low cohesion properties of the tested type of adhesives and directing to a relatively low degree of crosslinking. Drug candidates of good solubility in the adhesives like lidocaine, benzoic acid and ibuprofen, had significantly lower values of G′ at 0.1 rad/sec than their placebos, with the G′ decreasing proportionally to increasing drug concentration. Only acrylic films loaded with nicotinic acid passed both Chu’s criteria.

**Fig. 6:**
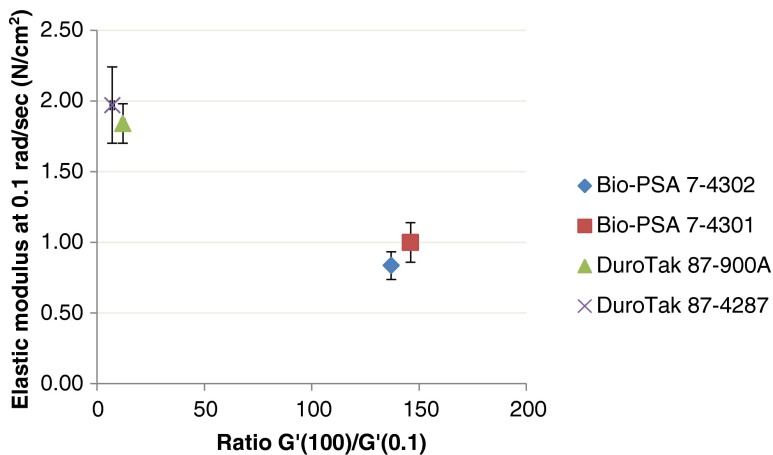
Differences in (**a**) the adhesive (G′ at 0.1 rad/sec) and (**b**) cohesive [G′(100)/G′(0.1)] properties between tested silicone and acrylic PSAs.

**Fig. 7:**
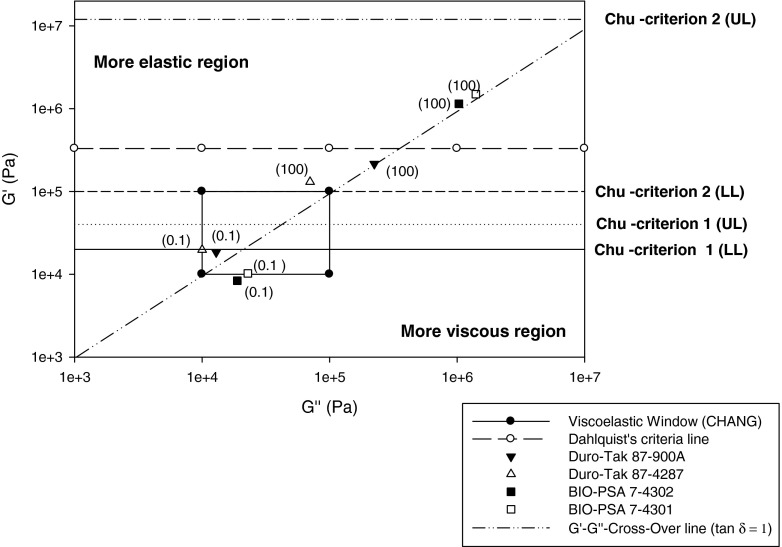
Moduli of tested drug-free pressure sensitive adhesives (mean, *n* = 3); *in brackets* frequency ω in rad/sec; *UL* upper limit; *LL* lower limit.

**Fig. 8:**
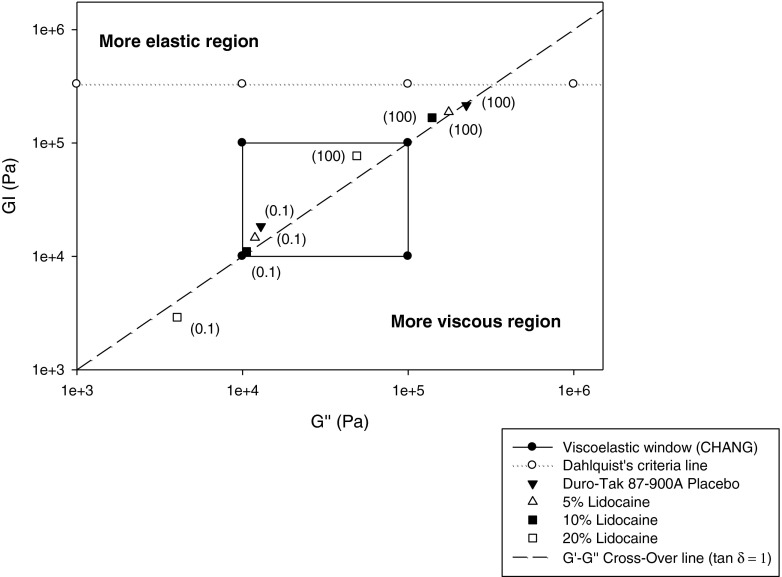
Moduli of Duro-Tak 87-900A at different nominal lidocaine concentrations (mean, *n* = 3); *in brackets* ω in rad/sec.

**Fig. 9:**
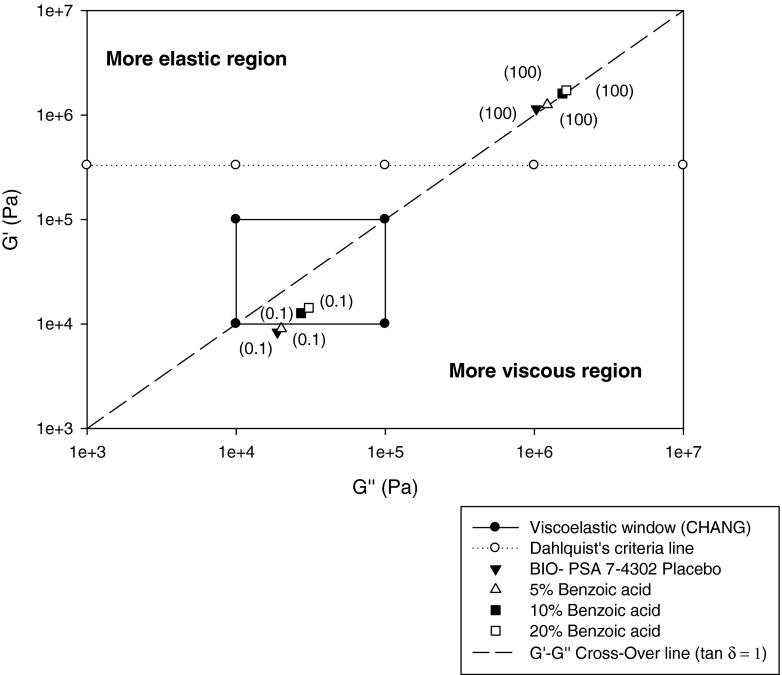
Moduli of Bio-PSA 7-4302 at different nominal benzoic acid concentrations (mean, *n* = 3); *in brackets* ω in rad/sec.

**Fig. 10:**
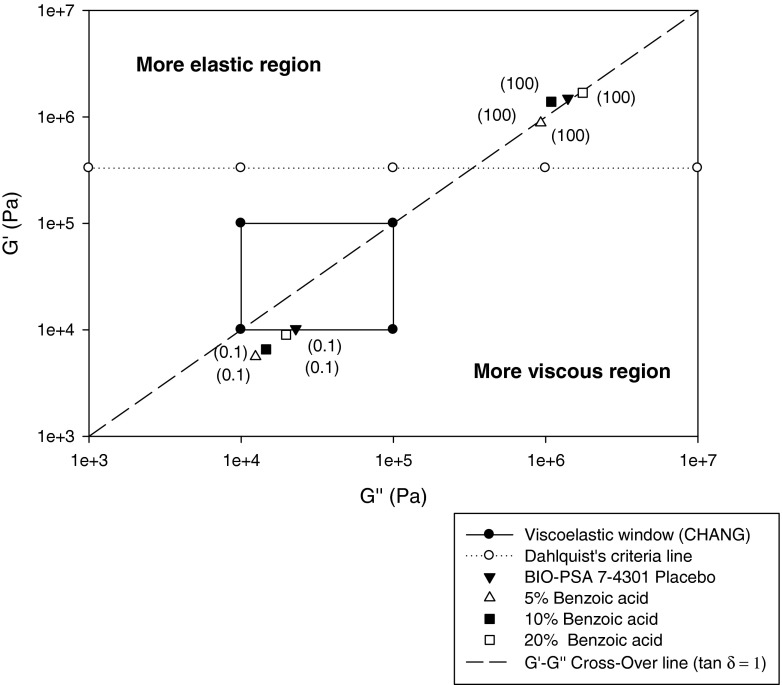
Moduli of BIO-PSA 7-4301 at different nominal benzoic acid concentrations (mean, *n* = 3); in brackets: ω in rad/sec.

All Bio-PSA 7-4301 films, including placebo, failed the Chu’s criterion of G′(0.1 rad/sec). Considering that Bio-PSA 7-4302 and Bio-PSA 7-4301 differ only in their solvent, observed differences in G′ between these two PSAs can be attributed to solvent effects; use of n-heptane apparently caused a decrease in elastic moduli compared to ethyl acetate, in tested samples. The moduli of the four PSAs are shown in Fig. 6 and differences between the two BIO PSA types are further illustrated in Fig. 7. Figure 8 shows that an increase of lidocaine concentration in 87-900A shifted the moduli to the lower left corner of the VW coordinate. Shift to the opposite direction (i.e. to the upper right corner) was shown by benzoic acid in Bio-PSA 7-4302 (Fig. 9). Bio-PSA 7-4302 films showed a different trend in the shift of moduli compared to Bio-PSA 7-4301 films. Moduli of up to 10% w/w of any model drug in Bio-PSA 7-4301 shifted to the lower left corner while the opposite was observed for Bio-PSA 7-4302. This “mixed response” where a 5% w/w load caused a slight shift to the lower left of the placebo was typical of the Bio-PSA 7-4301 formulations as shown in Fig. 10.

## DISCUSSION

### Correlation Between Solubility Parameters of Drug and Monomer PSA Units, with Saturation Status and Viscoelastic Properties of the Films

Inherently, the silicone-type PSAs have lower solubility parameters as compared to the acrylic-type PSAs. Both the dimethyl siloxane (DMS) and the resin for DMS (MQ) have similar solubility parameters i.e. 15.10 and 15.48 MPa^1/2^ respectively. They should affect the solubility parameter of the polymer quite similarly with respect to a resin:polymer ratio of 55:45 typical for Bio-PSA 7-4300 amine compatible silicone-type PSAs (17). Comparing the solubility parameter of the main monomer of acrylic-type PSA (2-ethylhexyl acrylate) to the silicone-type PSA’s monomer units, the acrylic-type PSA’s main monomer solubility parameter is about three units higher (15.10 and 15.48 MPa^1/2^ compared to 18.86 MPa^1/2^).

Drug solubility in the adhesive film was dependent on the difference in solubility parameter between the drug and the adhesive polymer units (Table III). The amount of undissolved drug reflected the difference between drug solubility and drug load as shown by benzoic acid in Duro-Tak 87-900A and Duro-Tak 87-4287; at 5% w/w load, benzoic acid was soluble in both adhesives whereas at 10% w/w load, benzoic acid was soluble only in Duro-Tak 87-900A and at 20% w/w it was suspended in both Duro-Taks).

Lidocaine, ibuprofen and benzoic acid were soluble in acrylic polymers, particularly in Duro-Tak 87-900A, because of the small difference in the drug/adhesive monomer solubility parameters; whereas the same drugs were suspended in the silicone adhesives due to a large δ difference. Nicotinic acid was suspended at all tested concentrations in both the acrylic-type (Duro-Tak) and silicone-type (Bio-PSA) adhesives due to a large δ difference.

Our results indicate a clear influence of total solubility parameter and saturation status of drug loaded films, on the viscoelastic properties of the films (Figs. 2 and 3). Subsaturated films showed lower moduli than the neat adhesive (placebo), directed to a plasticizing effect. As lidocaine is the model drug with the lowest solubility parameter it can be used to illustrate the effect of the difference in solubility parameter between drug and acrylic- or silicone-type PSA respectively. By increasing the lidocaine concentration, all three moduli (G′, G″, G*) and complex viscosity decreased in Duro-Tak 87-900A and Duro-Tak 87-4287 but not in the Bio-PSAs where the opposite was shown. The silicone-type PSAs have lower solubility parameters compared to the acrylic-type PSAs, which means that the solubility parameter difference between lidocaine and silicone-type PSA is greater than the difference between lidocaine and acrylic-type PSA. This difference is big enough to show a very different rheological behaviour between lidocaine-loaded acrylic-type PSAs and lidocaine-loaded silicone-type PSAs, in agreement with different drug solubilities in tested adhesives. Films with suspended drug crystals tended to show higher moduli than the placebo’s, presumably due to the presence of undissolved solids. The observed correlation between physical state of drug and moduli of the adhesive can be used for a semi-quantitative estimation of drug solubility, as it can be e.g. illustrated by benzoic acid in Duro-Tak 87-900A: Initial decrease of G′ by drug loading is followed by an increase of G′ at a drug load above 10% w/w. The resulting U-shaped G′ profile is consistent with the microscopic analysis showing that at 5% and 10% w/w loading the films were subsaturated whereas at 20% w/w there was visible drug crystallisation. Corresponding estimates of drug solubilities in the acrylic adhesives from viscoelastic data are shown in Table IV; the estimated solubility ranges correlated well with the microscopic observations, confirming that viscoelastic patterns are sensitive to the saturation status of the films.

**Table IV:** Drug Solubility Ranges Estimated from Rheological and Microscopic Data in Comparison to Drug Solubility Predicted by the Flory Equation (see also Table III). Drug Load of Duro-Tak Adhesives is Corrected for Measured Mean Solvent Residues in Pure Adhesives

PSA	Model drug	Drug solubility estimate from rheological data (% w/w)	Drug solubility estimate from microscopic data (% w/w)	Drug solubility estimate from limiting form of Flory equation (% w/w)
87-900A	Lidocaine	> 22.5	≥ 22.5	23.7
	Ibuprofen	> 22.5	≥ 22.5	19.4
	Benzoic acid	11.2–22.5	< 11.2	7.9
	Nicotinic acid	< 5.6	< 5.6	1.5
87-4287	Lidocaine	> 21.0	< 21.0	23.7
	Ibuprofen	< 22	< 22	19.4
	Benzoic acid	< 10.5	< 10.5	7.9
	Nicotinic acid	< 5.2	< 5.2	1.5
Bio-PSA	Lidocaine	< 5	< 5	3.1
	Ibuprofen	< 5	< 5	2.4
	Benzoic acid	< 5	< 5	0.9
	Nicotinic acid	< 5	< 5	0.1

The microscopic observations were also in congruence with the calculated estimates of drug solubility according to Flory’s equation (Table II). For example, the calculated drug solubilities in silicone PSAs were all lower than 5% w/w, in agreement with microscopic observations; in acrylic PSAs all calculated values were within the drug solubility concentration ranges that were observed via microscopy. Therefore, the limiting form of Flory’s equation was shown to be a useful tool for the calculation of drug solubility in silicone and acrylic drug-in-adhesive films, based on solubility parameter differences.

Deviations from microscopic results and estimates based on solubility parameters of drug and monomer PSA units can be anticipated when plasticizing effects of molecularly dispersed drug amounts are dominating over any cohesion strengthening effects exerted by suspended drug crystals. Such situation may explain results obtained on samples with 5% w/w nicotinic acid, where moduli with suspended drug crystals were statistically similar to their corresponding placebos (Fig. 2a and b), and on Duro-Tak 87-4287 samples with 20% lidocaine, where the modulus was lower than 10% lidocaine although sporadic crystals were already detectable in these films (Fig. 2b). Accordingly, drug solubility estimates from rheological measurements have to be interpreted with care, especially when drug solubility is similar to tested drug concentration as demonstrated for 5% w/w nicotinic acid and 20% w/w lidocaine.

Cold flow of the adhesive during storage can compromise the smooth removal of the patch from the primary package. During application to the skin, creeping of the drug loaded adhesive might lead to “black rings” surrounding the patch and/or to patch adherence to clothes covering the application site. Measured creep compliance data was in congruence with the frequency sweep results, directing to increased cold flow for subsaturated drug-in-adhesive films and decreased cold flow for films containing suspended drug particles, compared to placebo films (Fig. 4).

### Viscoelastic Parameters as Assessment Criteria for Adhesive Performance

Tack or bonding, requires low elastic (storage) and viscous (loss) shear moduli to enable the material to adequately deform, flow and adhere on skin surfaces of different contours and texture. Considering that Young’s modulus of skin was reported to be between 0.1 and 0.3 MPa, the elastic modulus of the patch should be of the same order to allow skin to move in a physiological way at the application site (20). In contrast, peel or debonding requires high elastic shear—and viscous moduli at high frequencies, to enable the removal of the material without cohesive failure from the skin surface (21). Figure 7 depicts elastic and viscous moduli of tested drug free adhesives measured at two different frequencies in relation to published fundamental viscoelastic requirements for pressure sensitive adhesives, considering target performance in terms of tack, shear and peel properties.

It is evident that all four drug-free adhesives have a bonding modulus G′ at 0.1 rad/sec much below the Dahlquist criteria line (shear modulus G′ = 330,000 Pa), as to be anticipated for materials that are “contact efficient” or tacky.

The G′-G″ cross-over line separates regions where the elastic modulus G′ is greater (i.e. tan δ <1) or smaller (tan δ >1) than the viscous modulus G″. As illustrated, bonding moduli measured at 0.1 rad/sec are located in the more elastic region for the acrylic systems, and in the more viscous region for the silicone adhesives. Overall, all values at low frequency measured at 32°C comply with or are very close to the viscoelastic window proposed by Chang (8) for “general purpose” PSAs, with G″ and G′ values falling into a region of 10^4^ to 10^5^ Pa at 0.01 rad/sec and 100 rad/sec. At high frequency, moduli were clearly lower for the acrylic adhesives remaining below the Dahlquist’s criteria line, in contrast to the high tack BIO-PSAs 4302/4301. However, despite the much higher G″ and G′ values the silicone adhesives still meet the 2nd Chu criterion for the upper and lower G′ value calculated based on the G′ ratio at low and high frequencies and the optimum G′ range at low frequency. Note: According to Chu’s 2nd criterion the ratio [G′ (at ω = 100 rad/sec)/G′ (at ω = 0.1 rad/sec)] equals 5 to 300. I.e. lower limit = (5 × 20,000 Pa) = 100,000 Pa and upper limit = (300 × 40,000 Pa) = 12,000,000 Pa.

All tested drug-free adhesives are slightly below the 1st Chu criterion, suggesting that moduli might have to be slightly increased to achieve optimum target of shear, tack and peel properties.

The adhesive/cohesive balance of the test material can be generally evaluated according to its location relative to the central viscoelastic window (VW) defined by Chang, considering that the elastic modulus (G’) reflects the strength against deformation and that the viscous modulus (G″) is indicative of dissipation i.e. flow of the material. According to Chang the G″/G′ is subdivided into four quadrants with a central one (only depicted in Fig. 7) representing the following material properties:

- The left quadrant of high G′ and low G′, can be assigned to materials without PSA properties.
- The right upper quadrant characterized high G′ and G″ values is typical for PSAs of high shear strength.
- The right lower quadrant can be attributed to PSAs of low cohesive strength and high dissipation which is favourable to cold temperature PSAs requiring good flow properties at low temperatures
- The left lower quadrant of can be assigned to removable PSAs of low elastic and viscous modulus.
- As mentioned the central quadrant corresponds to general purpose PSAs, described by medium cohesive strength and dissipation properties.

Chu’s criteria lines illustrate the G′ corridors found for hydrocarbon rubber-based adhesives with an optimum balance of tape properties, i.e. shear, peel, tack.

For example, drug loading of Duro-Tak 87900A with lidocaine leads to shift of the G″/G′ data pairs towards the lower left corner of the diagram indicating decrease of the cohesive strength (Fig. 8). A shift into the opposite direction (i.e. to the upper right corner) would accordingly mean increasing cohesive strength as shown by the benzoic acid in Bio-PSA 7-4302 and Bio-PSA 7-4301 (Figs. 9 and 10).

### Effect of Molar Drug Concentration on the Viscoelastic Properties of the Films

**Fig. 11:**
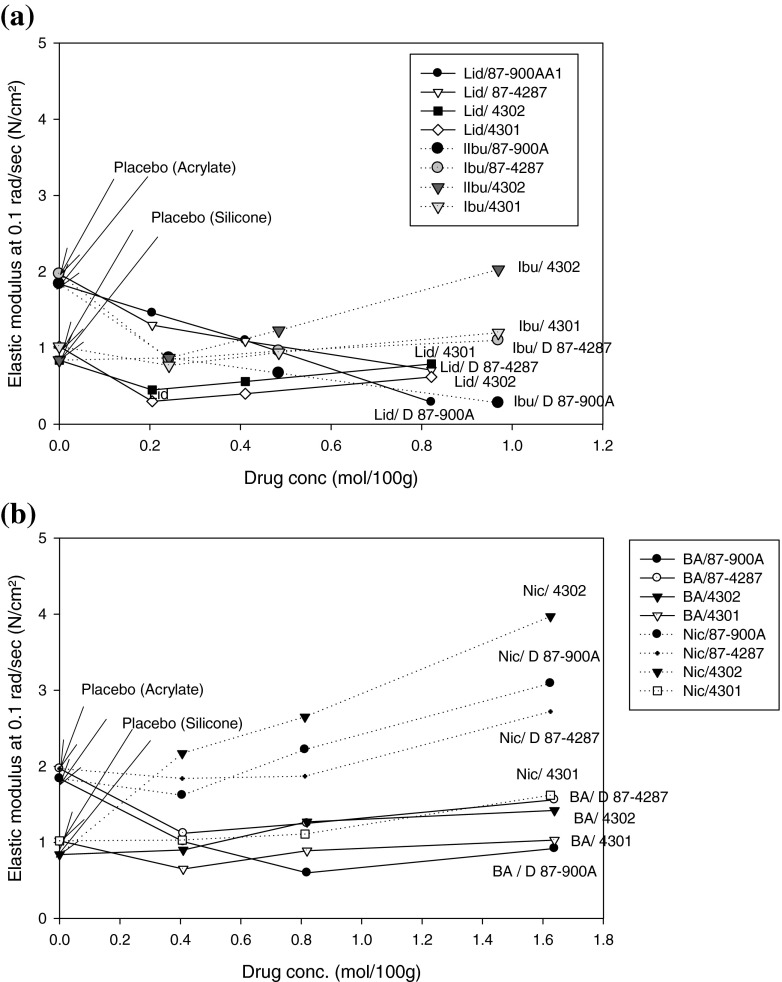
Elastic moduli of tested adhesives at different nominal molar drug loads, comparison of drugs with (**a**) similar (lidocaine and ibuprofen), (**b**) different solubility parameters (benzoic acid and nicotinic acid).

Figures 11a and b illustrate that drugs having quite similar solubility parameters (ibuprofen, lidocaine) also have quite similar elastic moduli at similar molar concentrations,and that drugs which are more different to this respect (benzoic acid, nicotinic acid) differ also more in their elastic moduli. Therefore, knowledge of the solubility parameter of the drug at the preformulation stage is useful to predict the rheological response to concentration changes in drug loaded films. The U-shape behaviour of benzoic acid in Duro-Tak 87-900A (Fig. 2a) can be attributed to its higher molar concentration at 20 wt.% load combined with a solubility parameter higher than lidocaine and ibuprofen which explains a lower solubility in the acrylic film.

The elastic moduli of nicotinic acid which are significantly different from the other drug-loaded acrylic PSAs and Bio-PSA 7-4302 represent rheological behaviour of these films containing actives with low solubility parameters.

In Bio-PSA 7-4301, nicotinic acid and ibuprofen exhibited the same elastic modulus at same molar concentrations; this was also observed for lidocaine and benzoic acid. Both silicone PSAs have the same chemical composition. The difference specified by the supplier is the solvent for the adhesive solids; heptane is used for Bio-PSA 7-4301 and ethyl acetate for Bio-PSA 7-4302. Therefore data are directing to a potential impact of solvents on adhesive performance of drug loaded matrix films. In tested silicone adhesives, dipole-dipole interactions with the drug and solvent molecules might have caused minor structural changes in the PDM/silicate network. However, to further explain the observed solvent-dependent shift of moduli in 4302 and 4301 type adhesives, further studies are needed which expand the scope of this investigation. Regardless of the described solvent impact, the effect of molar drug concentration on rheology was detectable also in the 4301 adhesive silicone adhesives, whereby the paired drugs, nicotinic acid/ibuprofen and lidocaine/benzoic acid, exhibited nearly identical viscoelastic moduli at the same molar concentrations.

### Correlation with Chu’s Criteria

It has to be considered that Chu’s criteria were based on results generated on blends of natural or synthetic rubber with tackifiers. Such tackifying ingredients are not per se needed for acrylic-type PSAs. Their adhesion/cohesion properties are varied by copolymer composition rather than by addition of a tackifying agent. Accordingly, extension of Chu’s criteria to other types of PSAs has to be done with caution (22). As shown, the tested acrylic adhesives are close to Chu’s criteria postulated for an optimum of peel, tack and shear properties, confirming the general approach based on G′ measurement at different frequencies also for this type of adhesive.

For silicone-type pressure adhesives a correlation between traditional tape properties and rheological data and consistency with Chu’s criteria has been already reported some years ago (9). Tested silicone adhesives had a lower G′ modulus at 0.1 rad/sec compared to the acrylic films. For example, the bonding modulus G′ can be increased by a slightly higher silicate resin portion in the reaction product with the PDMS polymer. As too high resin content would reduce tack properties, adjustment of the polymer/resin ratio has to be carefully tailored to the needs of the formulation. G′ values of 4302 and 4301 adhesives meet the 2nd criteria of Chu at high frequency, indicating good peel adhesion. Compared to the acrylics shear modulus G′(100 rad/sec) of the silicone adhesives is much higher, suggesting that moduli range at high frequencies can be relatively broad without compromising target optimum of ad-/cohesion properties when comparing different classes of adhesives.

### Impact of Adhesive Chemistry on the Viscoelastic Properties

Different rheological behaviour of acrylic and silicone based polymers as depicted in Fig. 7 can be also demonstrated by creep tests results, showing greater cold flow for the silicone PSAs.

The creep compliance of Duro-Tak 87-900A (13.2 × 10^−4^ Pa^−1^) was approximately three times that of Duro-Tak 87-4287 placebo (4.8 × 10^−4^ Pa^−1^) which means that Duro-Tak 87-900A will show higher cold flow than Duro-Tak 87-4287 under constant creep pressure. Comparing the acrylic-type PSA to silicone-type PSA, both silicone-type PSAs showed even greater creep compliance (32 × 10^−4^ Pa^−1^).

These observed differences in the viscoelastic properties between tested acrylic-type PSAs and silicone-type PSAs can be attributed to their different chemistry, reflecting basically different physico-chemical properties of carbon and silicone based adhesive polymers. For both types of adhesives adequate creep compliance can be adjusted by addition of cross-linking agents and/or appropriate solids which will reduce cold flow properties and increase resistance to deformation. For example, in the case of silicone-based polymers the silicate resin portion has to be optimized with respect to effects caused by selected drug load.

## CONCLUSION

Observed drug solubilities in the polymers were in agreement both with solubility parameter differences calculated for model compounds and adhesive monomers and the calculated volume fraction solubility using the limiting form of the well-known Flory equation as suggested by Fedors for estimation of water solubility in hydrocarbon polymers (19). As such, solubility parameter can be regarded as a powerful tool to predict drug solubility at an early development stage in those TDD systems for which specific molecular drug-polymer interactions can be neglected.

Drug suspensions typically caused an increase of the elastic modulus and decrease of creep compliance, dependent on the drug concentration of the adhesive film. An opposite effect was observed for solution matrices, where dissolved drug amounts were acting as softening agents. The observed impact of different load, solubility parameters and physical state of the selected compounds on viscoelastic properties of the adhesive has to be correspondingly considered when other ingredients like stabilising agents or skin penetration enhancers have to be added to the adhesive film. In addition, the effect of any concentration changes of drug and additives due to release to human skin must be taken into consideration in TDD formulation studies to ensure that the viscoelastic properties of the transdermal patch matrix are kept in a target range over the intended application period.

Described rheology studies are essential to specify an appropriate application window for viscoelastic parameters like elastic and viscous moduli for both raw materials and drug loaded formulations during development of TDD systems. Such specifications have to be supported by *in vivo* studies, which expand the scope of this study. Our investigations suggest that the known Chu criteria for rubber/resin based adhesives are useful also for acrylic- and silicone PSAs. Further research work in this field will show if and how these criteria and the CHANG application window have to be adapted for other adhesive formulations, different skin regions and conditions. In any case, physiological skin movement at the application site should not be compromised by too high elastic moduli of the patch formulation. Accordingly, this parameter has to be optimized together with other critical quality attributes, which are relevant for the adhesive performance like e.g. adhesive film thickness and type of backing.

## Abbreviations

DMS: dimethyl siloxane
LVR: linear viscoelastic region
PSA: pressure sensitive adhesive
TDD: transdermal drug delivery
VW: viscoelastic window
*G*′: elastic shear modulus
*G*″: viscous shear modulus
*J*: creep compliance
*δ*: solubility parameter
*ω*: angular frequency
